# Age-group visibility in WHO schistosomiasis reporting: decomposing preventive-chemotherapy coverage indicators, 2014–2024

**DOI:** 10.3389/fpubh.2026.1969131

**Published:** 2026-10-27

**Authors:** Xinrui Xu

**Affiliations:** College of Basic Medical Science, Dalian Medical University, Dalian, China

**Keywords:** adults, data representation, preventive chemotherapy, reproducibility, routine health data, schistosomiasis, school-age children, treatment coverage

## Abstract

**Background:**

Age-inclusive schistosomiasis policy requires monitoring that distinguishes between eligible age groups. The 2022 World Health Organization (WHO) guideline recommended treatment from 2 years of age in qualifying communities. We examined what public reporting makes visible for adults and school-age children (SAC), distinguishing information availability from programme scope and treatment receipt.

**Methods:**

We analysed all 567 country-year records from 52 countries during the 2014–2024 window in a frozen WHO schistosomiasis Preventive Chemotherapy and Transmission Control databank snapshot. Counts of adults requiring treatment and adults reported as treated were derived using a source-specific contract for interpreting age fields. Population-weighted adult and SAC reported-coverage indicators were each decomposed into a reporting-denominator share and conditional reported coverage. We audited missingness states, count anomalies, and prespecified exclusions and assessed fixed-country support and leave-one-country-out influence. No causal effect of the guideline was estimated.

**Results:**

In 2024, 83,126,427 of 134,806,681 SAC were reported treated (61.7%), compared with 17,368,705 of 118,864,858 adults (14.6%). The SAC indicator combined an 85.2% reporting-denominator share with 72.3% conditional reported coverage; the corresponding adult components were 47.7 and 30.6%. Of the adults represented in the denominator, 34.3% appeared in SAC-only records, potentially reflecting programme scope, and 18.0% in records without an age label. The adult indicator was lower in every year and in every sensitivity analysis. However, programme expectations were not the same throughout the study period: 2014–2021 preceded the broader guideline, whereas 2022–2024 represented the early calendar years following or coinciding with its publication. Logical missing-report envelopes placed the 2024 adult-minus-SAC difference between −61.8 and +5.3 percentage points, leaving the actual ordering of treatment coverage between adults and SAC unidentified.

**Conclusion:**

The reported adult–SAC difference combines the availability of valid age-specific treatment information with the treatment reported within supported records. A low reporting-denominator share cannot distinguish programme scope from incomplete reporting. Preschool-age treatment was not separately observable. Age-inclusive monitoring requires explicit age categories and documented eligibility and reporting states; these data do not identify verified uptake, individual equity, or causal guideline effects.

## 1 Introduction

Schistosomiasis remains a major neglected tropical disease in tropical and subtropical settings, where exposure to infested freshwater often coexists with constrained access to safe water, sanitation, diagnosis, and treatment (1). The World Health Organization (WHO) reports transmission in 79 countries and estimates that at least 253.7 million people required preventive treatment in 2024; approximately 93.9% of those requiring treatment lived in Africa (1). The estimated treatment requirement represents the number of people needing preventive chemotherapy, rather than the number of diagnosed infections. The present study examines global reporting, covering all country-year records available within the selected WHO database window, with most included countries in the African Region. Preventive chemotherapy with praziquantel is central to morbidity control and the 2021–2030 elimination agenda (1–3). Delivery and monitoring have historically concentrated on school-age children (SAC), for whom school-based treatment created a scalable platform and a consistently distinguished age category in global programme metrics.

The biological and programmatic relevance of age does not end with school. Adults can remain exposed through farming, fishing, irrigation work, and domestic contact with infested water. Eggs retained in tissues can produce chronic intestinal, hepatosplenic, urinary, or genital morbidity, while eggs excreted into freshwater can maintain transmission through snail hosts (1, 4). Where adults carry substantial infection burdens, untreated adult infections may sustain a community reservoir; transmission models show that the need for community-wide treatment depends on the local age distribution and intensity of infection, rather than age alone (5, 6). Adult participation also depends on delivery channels, work and opportunity costs, knowledge, perceived need, concerns about adverse effects, and whether community distributors reach the intended populations (7–10). In 2022, the WHO recommended annual preventive chemotherapy at 75% coverage or higher in all age groups from 2 years of age in eligible communities with schistosomiasis prevalence of 10% or higher (2, 11). This policy shift widened the population that programmes are expected to serve and gave greater urgency to an existing monitoring question: can existing reporting systems make implementation beyond SAC visible?

One aggregate coverage ratio cannot answer that monitoring question. Routine health information systems support repeated programme assessment, but their usefulness depends on completeness, internal consistency, representativeness, organizational processes, and use in decision-making (12–17). We define age-group visibility as the capacity of the public reporting system to distinguish an age group and link its treatment-requirement denominator to valid treatment information. We operationalize it through the reporting-denominator share and the partition of adult denominators by observable information state. Data equity refers here to the capacity to assess policy-relevant age groups on comparably explicit and auditable terms (15); it is a principle of data representation, not an additional numerical equity index. Reporting completeness concerns whether expected information is supplied, whereas programme scope concerns which groups a programme intends or is eligible to treat. Either may shape observed visibility, and the source labels alone cannot distinguish their contributions.

The WHO’s Preventive Chemotherapy and Transmission Control (PCT) databank compiles annual country reports through standardized reporting processes (18, 19). Such reports support global accountability, but programme-reported coverage is not equivalent to independently surveyed uptake. Across 214 neglected-tropical-disease mass drug administrations, routinely reported and surveyed estimates did not always lead to the same programme classification, and discrepancies have also been documented in schistosomiasis coverage surveys in Ethiopia, Zanzibar, and Mozambique (10, 20–23). A national intervention history in Ghana further showed why programme interpretation must account for changing survey designs, spatial aggregation, and the scale at which heterogeneity becomes visible (24); work in Sierra Leone similarly combined impact assessment with independent coverage monitoring (25).

These studies leave a measurement gap that can be addressed. A low global adult reported-coverage percentage can arise when only part of the valid adult population requiring treatment is represented in records containing valid adult treatment information, when reported treatment is low within those records, or when both conditions apply. Missing reports, changing denominators, implausible count relationships, and the weight of large countries add further ambiguity. These components raise different questions about programmes, yet their separate contributions are obscured when only the final ratio is reported.

Our research question was: to what extent do global WHO country reports permit characterization of reported schistosomiasis preventive-chemotherapy coverage among adults and SAC, and how much of each indicator reflects the availability of valid age-specific treatment information? We reconstructed and audited age-structured reporting from 2014 to 2024. The objectives were to: (i) compare population-weighted reported-coverage indicators among SAC, adults, and the total population requiring preventive chemotherapy; (ii) decompose both SAC and adult indicators into a reporting-denominator share and conditional reported coverage; and (iii) quantify sensitivity to missingness states, anomalous records, and country composition. The study provided a validated global reconstruction, a diagnostic interpretation of its headline ratios, and an auditable workflow. We prespecified the descriptive expectation that the adult reported-coverage indicator would remain below the SAC indicator. The 2014–2021 period provides a baseline under inherited SAC-centred recommendations, which also included selected at-risk adults; 2022–2024 are early calendar years at or after publication of broader recommendations (2, 11, 19). Adult eligibility and reporting expectations were therefore not constant throughout the study period. National adoption dates were unavailable, so guideline publication provided policy context rather than a clearly identifiable intervention. The source did not separately encode preschool-age treatment, allowing SAC/adult reporting to be assessed but preventing assessment of complete all-age coverage.

## 2 Materials and methods

### 2.1 Study design and reporting framework

This was a descriptive observational study using routinely collected, aggregate country-year reports. The unit of observation in the source data was the country-year, and the primary analysis window was 2014–2024. Global measures were ratios of summed counts and were therefore population-weighted; they should not be interpreted as the mean experience of an endemic country. The study reporting framework was guided by the REporting of studies Conducted using Observational Routinely-collected health Data (RECORD) extension for observational studies using routinely collected health data (26), the WHO coverage-monitoring guidance (27, 28), and the principle that routine information is useful for decision-making only when its technical definitions and data-production processes are explicit (16, 17).

No sampling-based hypothesis tests or confidence intervals were calculated. The workbook is a compilation of reports received by WHO rather than a probability sample from a defined superpopulation. The main sources of uncertainty were data completeness, field semantics, denominator revisions, count anomalies, and country composition. We represented these uncertainties through explicit denominators, missingness summaries, prespecified sensitivity analyses, and influence diagnostics rather than through sampling intervals.

### 2.2 Data source and frozen snapshot

Data came from the WHO PCT Databank—Schistosomiasis workbook, whose stated source is annual reports submitted by countries to WHO (19). The PCT system was developed to standardize the flow of epidemiological and implementation data from national programmes to WHO headquarters via its regional offices and to make country-level indicators available for programme planning and monitoring (18). WHO’s Joint Application Package has been used for annual country reporting since 2013 (19, 29).

The workbook was downloaded on 15 August 2026 and archived without manual modification. The analysis used the SCH_DATA worksheet of SCH_data.xlsx, which contained 1,004 country-year rows covering 58 countries over the period from 2006 to 2024. The primary 2014–2024 window contained 567 rows from 52 countries. Each country-year key was unique. The reviewer-only workbook contains the source cell values and a recoverable copy of the original WHO workbook, together with derived data, analysis outputs, analysis code, and technical verification information. File checksums are provided in the workbook documentation.

### 2.3 Country selection and analysis-specific exclusions

Country inclusion was determined entirely by record availability and calendar year. We retained every country-year record dated 2014–2024 in the frozen SCH_DATA worksheet, without restricting geographic coverage or requiring a treatment report. Filtering the 1,004 source records excluded 437 records dated before 2014 and retained 567 records representing 52 distinct countries. Six countries had source records only outside the primary window: Dominican Republic, Iraq, Libya, Oman, Saudi Arabia, and Suriname. The 52-country count represents the union of countries across the study period; it does not imply that every included country required preventive chemotherapy in 2024. China had records for 2014–2019 only; the annual source therefore contained 52 country records in 2014–2019 and 51 in 2020–2024. No country or record was excluded from the retained source dataset solely because treatment information or an age label was missing.

A record’s retention in the source dataset was distinguished from its contribution to a particular estimate. Missing, non-numeric, or negative count values did not contribute to the corresponding numerical sums. Non-numeric requirement states were retained as source information. A negative derived adult requirement was excluded from the adult denominator; calculation of an adult treatment ratio additionally required a positive, valid adult denominator, an explicit adult-inclusive label, and valid non-negative treatment components. Thus, SAC-only records and records without an age label did not supply an adult numerator, while their valid adult denominator values remained part of the primary adult denominator. Reported zeros were retained. Coverage above 100% was retained in the primary analysis and excluded only in the prespecified variants described below. The fixed-support analysis used a separate restriction to countries with valid age-specific denominators in all 11 years.

### 2.4 Age-field semantics and derivation of adult counts

The WHO’s schistosomiasis PCT page defines SAC as individuals aged 5 to < 15 years and adults as individuals aged 15 years or older (19). Its denominator model describes high-risk areas as requiring treatment for all SAC and adults, moderate-risk areas as requiring treatment for specified proportions of SAC and adults, and low-risk areas as requiring treatment for a proportion of SAC (19). Historical Joint Application Package documentation similarly separated preschool-age children, SAC, and adults, and described schistosomiasis target populations in terms of SAC and adults (29).

We treated adult subtraction as a source-specific reporting identity, not a demographic assumption. The adult population requiring preventive chemotherapy was calculated as:

$$A_{\mathrm{ct}}^{\mathrm{req}}=T_{\mathrm{ct}}^{\mathrm{req}}-S_{\mathrm{ct}}^{\mathrm{req}}$$

where $T_{\mathrm{ct}}^{\mathrm{req}}$ was the total population requiring preventive chemotherapy and $S_{\mathrm{ct}}^{\mathrm{req}}$ was the number of SAC requiring preventive chemotherapy for country c in year t . For an age-group record labelled SAC and Adults, adult treatment was:

$$A_{\mathrm{ct}}^{\mathrm{tx}}=T_{\mathrm{ct}}^{\mathrm{tx}}-S_{\mathrm{ct}}^{\mathrm{tx}}$$

For a record labelled Adults, total reported treatment was used directly as adult treatment. Records labelled SAC were not considered adult-inclusive. Missing age-group values remained missing rather than being inferred.

The semantic contract for the primary analysis window allowed only the non-missing labels Adults, SAC, and SAC and Adults. Any record labelled All caused the pipeline to stop because the residual could then include preschool-age children and would no longer identify adults alone. This safeguard is important after the 2022 recommendation expanded eligible treatment to age 2 years and older (2, 11). We did not estimate preschool-age treatment or coverage.

We externally triangulated the age-field interpretation against WHO’s 2024 Global Health Observatory summary (30). Exact reconstructed counts were required to yield estimates within 0.05 percentage points of the publicly displayed 61.7% SAC coverage and 14.6% adult coverage and to reproduce the reported 21 adult-reporting countries.

### 2.5 Missingness and anomaly contract

For every count field, missing values, reported zeros, and reported positive values were treated as distinct states. A missing treatment value was not changed into an observed zero because the absence of a submitted value could reflect no treatment, unreported treatment, or another reporting state. The annual reported-coverage estimands nevertheless credited only treatment counts actually reported: records without valid treatment information contributed valid denominator mass but no treatment count to the numerator. SAC treatment contributed when the SAC denominator was valid and SAC treatment was reported as zero or positive. Adult treatment contributed only when the age group was explicitly adult-inclusive, the adult denominator was positive, and the requirement and treatment counts were arithmetically valid.

To interpret the absence of adult treatment information without assuming its cause, we partitioned valid adult denominator mass into four observable reporting states: valid adult treatment information; an adult-inclusive label with missing or invalid treatment information; a SAC-only label; and a missing or unresolved age label. These states describe the source record, not programme eligibility, delivery, or treatment receipt. In particular, a SAC-only label may reflect a programme targeted at SAC under the applicable eligibility rules, rather than failure to report an adult programme. A missing age label represents unresolved source information; it does not establish that eligible adults received no treatment.

The workflow preserved source values and stopped if the prespecified anomaly set changed. A negative derived adult denominator was retained and flagged but excluded from adult arithmetic. A record with positive derived adult treatment and a zero adult denominator was also retained and flagged but excluded from adult coverage. Source programme-coverage values and derived adult-coverage values above 100% were not capped. They remained in the primary preserved-source analysis and were separately excluded in sensitivity analyses.

### 2.6 Estimands

For each year, SAC reported coverage was:

$$C_{t}^{S}=100\times \frac{\sum_{c}J_{\mathrm{ct}}S_{\mathrm{ct}}^{\mathrm{tx}}}{\sum_{c}S_{\mathrm{ct}}^{\mathrm{req}}}$$

Adult reported coverage was:

$$C_{t}^{A}=100\times \frac{\sum_{c}I_{\mathrm{ct}}A_{\mathrm{ct}}^{\mathrm{tx}}}{\sum_{c}A_{\mathrm{ct}}^{\mathrm{req}}}$$

where $I_{\mathrm{ct}}=1$ denoted a valid adult-inclusive treatment report. The denominator included all valid counts of adults requiring preventive chemotherapy, whereas the numerator included treatment only from valid adult-inclusive reports. Overall reported coverage was the sum of reported valid total-treatment counts divided by the full valid denominator for the total population requiring treatment. The adult–SAC reported-indicator difference was $C_{t}^{A}-C_{t}^{S}$ , in percentage points.

Adult reported coverage was decomposed as:

$$C_{t}^{A}=R_{t}^{A}\times Q_{t}^{A}/100$$

where adult reporting-denominator share was:

$$R_{t}^{A}=100\times \frac{\sum_{c}I_{\mathrm{ct}}A_{\mathrm{ct}}^{\mathrm{req}}}{\sum_{c}A_{\mathrm{ct}}^{\mathrm{req}}}$$

and conditional adult reported coverage was:

$$Q_{t}^{A}=100\times \frac{\sum_{c}I_{\mathrm{ct}}A_{\mathrm{ct}}^{\mathrm{tx}}}{\sum_{c}I_{\mathrm{ct}}A_{\mathrm{ct}}^{\mathrm{req}}}$$

SAC reported coverage was decomposed symmetrically. With $J_{\mathrm{ct}}=1$ for a valid SAC treatment report,

$$C_{t}^{S}=R_{t}^{S}\times Q_{t}^{S}/100$$

where $R_{t}^{S}=100\times \sum_{c}J_{\mathrm{ct}}S_{\mathrm{ct}}^{\mathrm{req}}/\sum_{c}S_{\mathrm{ct}}^{\mathrm{req}}$ and $Q_{t}^{S}=100\times \sum_{c}J_{\mathrm{ct}}S_{\mathrm{ct}}^{\mathrm{tx}}/\sum_{c}J_{\mathrm{ct}}S_{\mathrm{ct}}^{\mathrm{req}}$ . Both decompositions were deterministic identities, not causal mediation models. Reporting-denominator share described the proportion of valid age-specific denominator mass linked to valid treatment reports; it did not prove programme eligibility, geographic reach, individual access, or treatment receipt.

In plain terms, the reporting-denominator share is the percentage of the age-specific requirement denominator that can be linked to a valid treatment count in this source. Its complement is a mixture of observable states, including SAC-only programme labels and unresolved reporting, rather than a measured reporting-failure rate. Conditional reported coverage is the reported treatment count divided by the requirement denominator within those linked records.

For deliberately extreme missing-report stress tests, treating the observed reported counts and valid denominators as fixed, we calculated upper envelopes that assumed every person represented in the valid age-specific denominator but lacking a valid treatment report had been treated. Lower values were the observed reported-coverage indicators. The corresponding logical adult-minus-SAC range extended from the adult lower value minus the SAC upper value to the adult upper value minus the SAC lower value. These quantities were not confidence intervals, plausible estimates, or formal bounds on true treatment receipt when administrative numerators or denominators might contain errors; they showed only that incomplete reporting did not establish which age group had higher treatment coverage.

The prespecified measures and their interpretation boundaries are summarized in Table 1.

**Table 1:** Prespecified measures and interpretation boundaries.

Measure	Calculation	Supported interpretation	Not supported
SAC reported-coverage indicator	Σ valid reported counts of SAC treated / Σ valid counts of SAC requiring PC	Population-weighted SAC treatment visible relative to the full valid SAC requirement denominator	Verified SAC uptake or biological effect
Adult reported-coverage indicator	Σ valid reported counts of adults treated / Σ valid derived counts of adults requiring PC	Population-weighted adult treatment visible relative to the full valid adult requirement denominator	Verified adult uptake, preschool-age coverage, or individual access
Adult–SAC reported-indicator difference	Adult indicator − SAC indicator	Descriptive age difference in global reported indicators	Identified treatment-receipt difference, individual equity, or causal discrimination
Age-specific reporting-denominator share	Σ age-specific denominator attached to valid treatment reports / Σ valid age-specific denominator	How much denominator mass is attached to valid treatment information	Geographic programme reach or verified eligibility
Conditional age-specific reported coverage	Σ reported treatment counts in supported records / Σ denominator in supported records	Reported treatment conditional on valid treatment-report support	Treatment uptake among sampled individuals
Logical missing-report envelope	(reported treatment count + denominator lacking valid treatment information) / valid denominator	Extreme upper stress test under universal treatment outside supported records	Confidence interval, plausible estimate, or imputation

SAC, school-age children; PC, preventive chemotherapy. Σ denotes summation across valid country-year records.

### 2.7 Sensitivity and influence analyses

Four prespecified variants were computed for every year: (i) the primary analysis preserving valid source values; (ii) exclusion of the entire country-year record when the source-supplied programme-coverage field exceeded 100%; (iii) exclusion of the entire country-year record when derived adult coverage exceeded 100%; and (iv) exclusion of the entire country-year record for the adjudicated adult arithmetic anomalies. Each exclusion therefore removed the affected record from all age-specific numerators and denominators. Variants were not selected according to the direction or perceived statistical favourability of their results. A fixed-support analysis additionally retained the 48 countries with valid SAC and adult denominators in every year from 2014 through 2024.

Country composition was examined through leave-one-country-out adult reported coverage. For each year, the population-weighted adult reported indicator was recomputed after removing one country at a time. An observation was flagged when removal changed the indicator by at least 2 percentage points, an absolute difference readily visible in programme summaries, or by at least 10% relative to the full aggregate, which remained informative at low values of the adult indicator. Continuous changes were retained for every country, and flagged countries remained in the primary analysis.

### 2.8 Reproducibility and software

The analysis was implemented in R with a project-local package lockfile. A targets pipeline linked the immutable workbook to derived data, annual summaries, sensitivity analyses, influence diagnostics, figures, and a Quarto reproducibility report. Automated tests asserted the source hash and schema, unique keys, missing-versus-zero behaviour, the exact anomaly set, permitted age labels, absence of All records, the 2024 WHO anchors, the symmetric decomposition identities, the adult information-state partition, and fixed-support behaviour. Archived reproduction records document a clean-room run under R 4.5.3 with 76 locked packages, execution of all tests and 31 targets from scratch, and byte-for-byte agreement of 12 designated core comma-separated values (CSV) and JavaScript Object Notation (JSON) outputs. The reviewer-only workbook provides the analysis code, environment specifications, numerical checks, and reproduction instructions.

### 2.9 Ethics

Ethical review and approval were not required. This study is a secondary analysis of publicly available, aggregate country-year programme data and involved no contact with human participants or access to identifiable individual-level data.

## 3 Results

### 3.1 Source support, age semantics, and data quality

The primary dataset contained 567 country-year records from 52 countries. The complete country list is given below in alphabetical order, with country names retained as recorded in the source. Among non-missing age-group values, 243 records were labelled SAC and Adults, four were labelled Adults, and the remaining non-missing records were labelled SAC. No primary-window record was labelled All; 247 records were therefore explicitly adult-inclusive by label. After validity checks, the annual number of adult-inclusive treatment reports ranged from 13 to 26.

Angola; Benin; Botswana; Brazil; Burkina Faso; Burundi; Cambodia; Cameroon; Central African Republic; Chad; China; Congo; Côte d’Ivoire; Democratic Republic of the Congo; Egypt; Equatorial Guinea; Eritrea; Eswatini; Ethiopia; Gabon; Gambia; Ghana; Guinea; Guinea-Bissau; Indonesia; Kenya; Lao People’s Democratic Republic; Liberia; Madagascar; Malawi; Mali; Mauritania; Mozambique; Namibia; Niger; Nigeria; Philippines; Rwanda; Sao Tome and Principe; Senegal; Sierra Leone; Somalia; South Africa; South Sudan; Sudan; Togo; Uganda; United Republic of Tanzania; Venezuela (Bolivarian Republic of); Yemen; Zambia; and Zimbabwe.

The source-semantic audit passed all 15 prespecified checks. For 2024, 253,671,539 people in the total population requiring preventive chemotherapy minus 134,806,681 SAC yielded 118,864,858 adults. Likewise, a total of 100,495,132 people reported treated minus 83,126,427 SAC reported treated yielded 17,368,705 adults. These counts produced 61.663% SAC-reported coverage and 14.612% adult-reported coverage and matched WHO’s published aggregation of 61.7%, 14.6%, and 21 adult-reporting countries (30). This concordance verified the implementation of WHO’s aggregation logic; it did not validate the underlying country reports as accurate records of treatment receipt.

One country-year (São Tomé and Príncipe, 2016) had a negative derived adult denominator. Six records had positive derived adult treatment with a zero derived adult denominator: Brazil in 2016 and 2022, and Cambodia, Egypt, Indonesia, and the Philippines in 2014. No record had negative derived adult treatment. Twenty-three country-years had source programme coverage above 100%, and nine had derived adult coverage above 100%. These values were preserved and flagged; invalid adult ratios were excluded according to the fixed anomaly contract.

### 3.2 Age-group differences before and after guideline publication

The adult reported-coverage indicator was lower than the corresponding SAC indicator in every year from 2014 through 2024 (Table 2; Figure 1). The adult indicator ranged from 8.29% in 2014 to 19.56% in 2016, whereas the SAC indicator ranged from 43.18% in 2014 to 70.84% in 2017. The reported adult-minus-SAC difference ranged from −29.44 percentage points in 2020 to −53.96 percentage points in 2017. The 2014–2021 observations describe an inherited SAC-centred reporting architecture with selected at-risk adults also eligible under earlier recommendations; they do not assume universal adult eligibility. The 2022–2024 observations are early calendar years at or after guideline publication, not a completed post-guideline implementation period. Both SAC and overall reported coverage fell in 2020 and remained below their 2019 levels in 2021, but the series was not treated as a causal estimate of COVID-19 disruption or policy change (31).

**Table 2:** Global reported-coverage indicators and symmetric decomposition, 2014–2024.

Panel A. Reported-coverage indicators and reporting counts						
Year	Overall reported, %	SAC reported, %	Adult reported, %	Adult–SAC, pp	Adult valid reporters	Missing total reports
2014	25.37	43.18	8.29	−34.89	13	20
2015	34.12	49.14	16.19	−32.95	23	15
2016	43.54	64.33	19.56	−44.76	23	13
2017	46.40	70.84	16.88	−53.96	24	12
2018	42.01	61.98	18.20	−43.78	22	15
2019	44.89	66.90	18.06	−48.85	19	14
2020	32.24	45.41	15.97	−29.44	23	16
2021	31.87	46.85	14.18	−32.68	25	12
2022	35.11	51.91	17.62	−34.29	26	15
2023	35.81	56.48	12.04	−44.45	21	17
2024	39.62	61.66	14.61	−47.05	21	16

Panel B. Symmetric decomposition components				
Year	SAC reporting share, %	SAC conditional, %	Adult reporting share, %	Adult conditional, %
2014	88.82	48.62	30.55	27.14
2015	86.14	57.05	63.56	25.47
2016	91.64	70.20	67.37	29.04
2017	95.37	74.28	56.79	29.72
2018	85.14	72.80	44.11	41.26
2019	88.31	75.76	44.40	40.67
2020	72.02	63.05	47.22	33.81
2021	84.16	55.67	69.92	20.28
2022	88.02	58.98	75.75	23.26
2023	87.37	64.65	64.88	18.55
2024	85.24	72.34	47.68	30.64

SAC, school-age children; pp, percentage points. Adult valid reporters are country-year rows with an adult-inclusive age label and valid adult treatment information. Missing total reports are country-year rows with missing or invalid total treatment information. Values are population-weighted ratios of summed counts, not country means.

**Figure 1:**
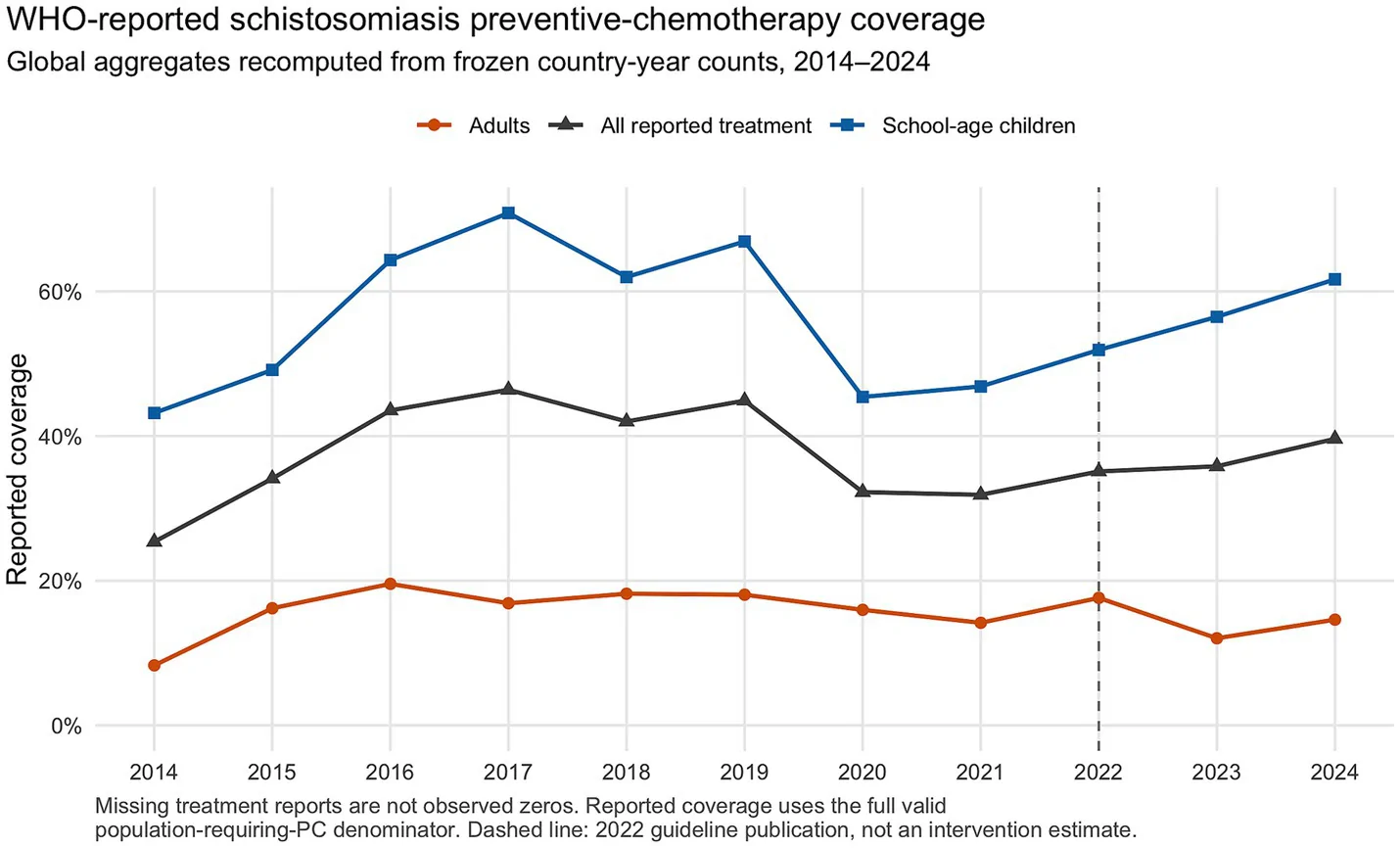
Population-weighted global schistosomiasis preventive-chemotherapy reported-coverage indicators by age group, 2014–2024. Lines show reported treatment counts relative to the full valid age-specific requirement denominators for school-age children (SAC), adults, and the total population. Missing values were not changed into observed zeros, but only submitted treatment counts entered the numerators. Values represent the World Health Organization (WHO) country reports and not independently verified uptake. The dashed marker at 2022 denotes guideline publication, not an identified intervention.

In 2024, 83,126,427 SAC were reported treated from a valid denominator of 134,806,681, giving a 61.66% reported-coverage indicator. Among adults, 17,368,705 were reported treated from a valid denominator of 118,864,858, giving 14.61%. Overall reported coverage was 39.62% (100,495,132/253,671,539), and the reported adult-minus-SAC difference was −47.05 percentage points. The WHO received valid total-treatment reports from 35 country records, of which 21 contained valid adult treatment information; 16 country records had missing total-treatment reports.

### 3.3 Symmetric decomposition and adult treatment-information states

Reporting support and conditional reported coverage varied separately over time and by age group (Table 2; Figure 2). Adult reporting-denominator share ranged from 30.55% in 2014 to 75.75% in 2022, compared with 72.02 to 95.37% for SAC. Conditional adult reported coverage ranged from 18.55% in 2023 to 41.26% in 2018, compared with 48.62 to 75.76% for SAC. Consequently, the age-group difference in the final indicator combined different proportions of the denominator linked to valid treatment information with different levels of reported treatment within those linked records. The first component did not distinguish programme scope from reporting completeness.

**Figure 2:**
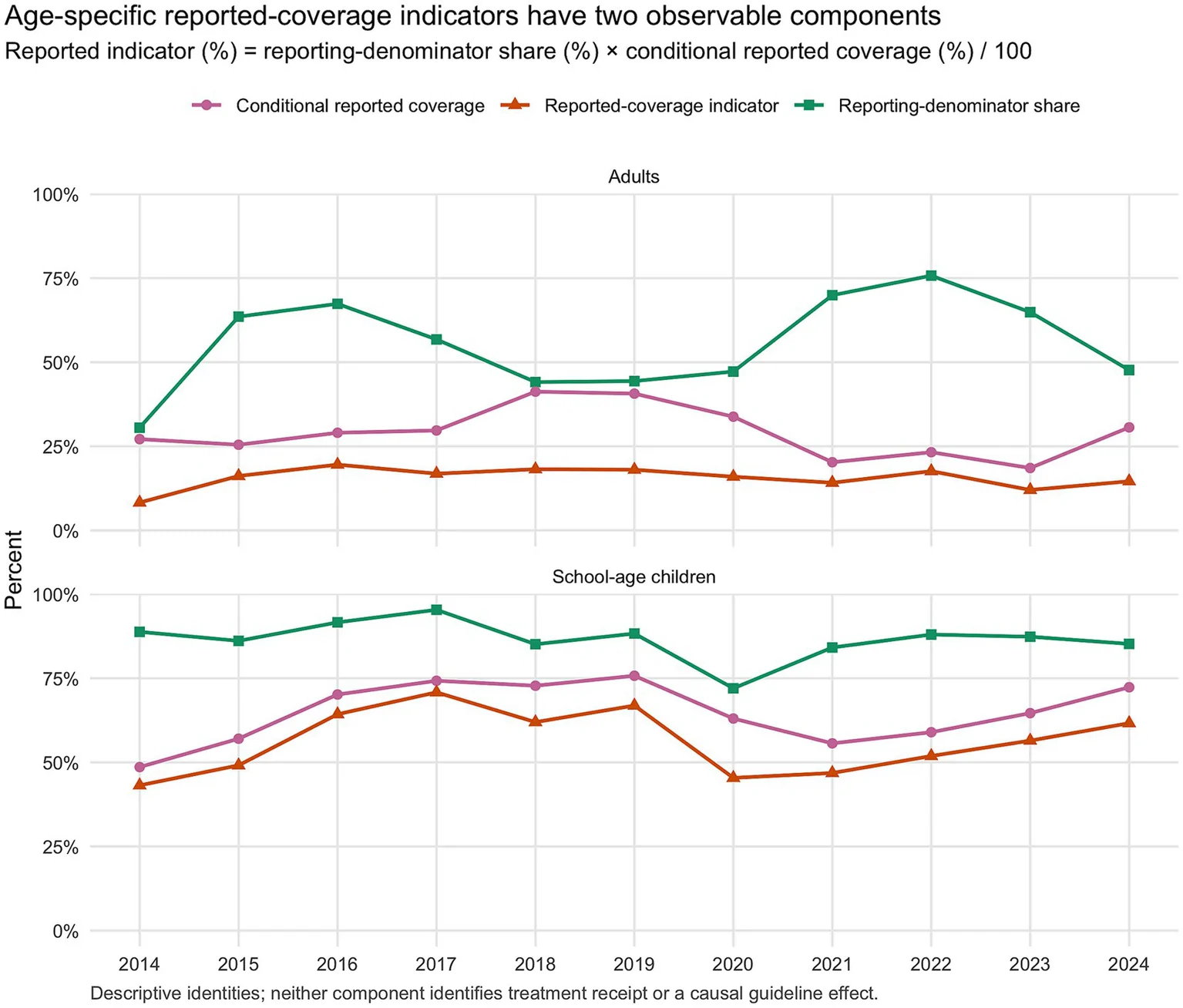
Symmetric decomposition of school-age children (SAC) and adult reported-coverage indicators, 2014–2024. For each age group, reported indicator (%) = reporting-denominator share (%) × conditional reported coverage (%) / 100. The identities are descriptive and should not be interpreted as causal mediation, geographic programme reach, or treatment uptake. For example, the 2024 adult components give approximately 47.7% × 30.6% / 100 ≈ 14.6%; SAC components give approximately 85.2% × 72.3% / 100 ≈ 61.7%. Exact identities use the unrounded values. Reporting-denominator share measures linked treatment information; it can reflect programme scope as well as reporting completeness.

In 2024, valid SAC treatment reports were attached to 114,909,331 of 134,806,681 SAC requiring preventive chemotherapy, a reporting-denominator share of 85.24%; conditional reported coverage was 72.34%. Valid adult treatment reports were attached to 56,680,632 of 118,864,858 adults requiring preventive chemotherapy, a reporting-denominator share of 47.68%; conditional reported coverage was 30.64%. Using unrounded values, both products reproduced the corresponding reported-coverage indicators within floating-point tolerance (61.66% for SAC and 14.61% for adults after rounding).

A simple reading of the 2024 adult calculation is useful. Of the 118.9 million adults in the valid requirement denominator, 56.7 million were in records with valid adult treatment information. Those records reported 17.4 million adults treated. The share with linked treatment information was therefore approximately 56.7/118.9, or 47.7%, and conditional reported coverage was approximately 17.4/56.7, or 30.6%. Using the original unrounded counts, the product equals 17,368,705/118,864,858, or 14.6% after rounding. Equivalently, per 100 adults represented by the denominator, records covering approximately 47.7 adults supplied valid adult treatment counts, and those records reported treatment corresponding to approximately 14.6 of the original 100 adults. This is denominator arithmetic, not a survey of 100 people. The same calculation for SAC gives 85.2% × 72.3% / 100 ≈ 61.7%.

The remaining valid adult denominator in 2024 was divided between 40,754,599 adults (34.29%) in 14 SAC-only records and 21,429,627 (18.03%) in 16 records with no age-group label (Table 3). No 2024 adult-denominator mass was attached to an adult-inclusive label with missing or invalid treatment information. The SAC-only category may reflect programme scope or eligibility under national practice, while the category without an age label leaves the reporting state unresolved. Neither category can be assigned wholly to reporting failure, and the database does not reveal whether adults were ineligible or untreated, whether their treatment went unreported, or whether their treatment information was lost during aggregation.

**Table 3:** Adult treatment-information states in 2024.

Observable source state	Country rows	Valid adult denominator	Share of valid adult denominator, %
Valid adult treatment information	21	56,680,632	47.68
Adult-inclusive label with missing or invalid treatment information	0	0	0.00
SAC-only age label	14	40,754,599	34.29
Age label not reported	16	21,429,627	18.03

SAC, school-age children. The categories partition the valid adult denominator of 118,864,858. They do not identify programme eligibility, treatment delivery, or treatment receipt. SAC-only records may reflect programme scope or eligibility and should not be interpreted uniformly as adult reporting failures.

The extreme upper reporting envelopes were 76.42% for SAC and 66.93% for adults. Combining lower and upper values placed the logical 2024 adult-minus-SAC difference between −61.81 and +5.26 percentage points. These limits are not plausible estimates or confidence intervals. They demonstrate that the ordering of actual treatment receipt was not identified by the public administrative compilation, even though the prespecified reported adult indicator was lower.

### 3.4 Sensitivity to source anomalies

The adult-minus-SAC reported-indicator difference remained negative in all 11 years under every prespecified sensitivity variant (review-only data file, worksheet Sensitivities); Table 4 displays the 2024 results. Excluding derived adult coverage above 100% reduced the adult indicator most visibly in the early years, from 8.29 to 5.98% in 2014, from 16.19 to 12.90% in 2015, and from 19.56 to 13.70% in 2016. It did not change the 2024 estimate because no 2024 derived adult coverage value exceeded 100%.

**Table 4:** Prespecified 2024 sensitivity and influence results.

Panel A. Prespecified data-quality sensitivities						
Analysis	Adult reported, %	SAC reported, %	Adult reporting share, %	Adult conditional, %	SAC reporting share, %	SAC conditional, %
Primary preserved-source analysis	14.61	61.66	47.68	30.64	85.24	72.34
Exclude source programme coverage > 100%	11.38	60.45	45.92	24.79	83.99	71.97
Exclude derived adult coverage > 100%	14.61	61.66	47.68	30.64	85.24	72.34
Exclude adjudicated adult arithmetic anomalies	14.61	61.66	47.68	30.64	85.24	72.34

Panel B. Leave-one-country-out influence			
2024 country omitted	Leave-one-out adult reported indicator, %	Change, pp	Relative change, %
Rwanda	11.88	−2.73	−18.70
Mozambique	16.18	+1.57	+10.74
Nigeria	16.18	+1.56	+10.70

SAC, school-age children; pp, percentage points. Complete 2014–2024 year-by-variant results are supplied in the review-only data file, worksheet Sensitivities. Countries are shown when omission changed the adult-reported indicator by at least 2 percentage points or by a relative change of at least 10%. No country was excluded from the primary analysis.

Excluding records whose source programme coverage exceeded 100% reduced the 2024 adult indicator from 14.61 to 11.38% and the SAC indicator from 61.66 to 60.45%. Exclusion of the adjudicated adult arithmetic anomalies did not alter the primary 2024 adult estimate because no adjudicated adult arithmetic anomaly occurred in 2024. The fixed-support analysis retained 48 countries with valid SAC and adult denominators in all 11 years; the adult indicator remained below the SAC indicator throughout, and the 2024 values were 14.61 and 61.68%, respectively. Thus, the sign of the reported-indicator difference was not generated by the entry or exit of countries with valid denominators, although its magnitude remained sensitive to source quality and country composition.

### 3.5 Country composition

Three countries crossed the prespecified influence threshold in the 2024 leave-one-country-out analysis. Removing Rwanda lowered adult reported coverage by 2.73 percentage points, from 14.61 to 11.88%, because its adult report contained a large adult denominator with reported treatment equal to that denominator. Removing Mozambique increased the global adult estimate by 1.57 percentage points (10.74% relative), and removing Nigeria increased it by 1.56 percentage points (10.70% relative). These diagnostics show that the global population-weighted result is sensitive to country composition; they do not justify excluding any country or establish why a national value was reported.

## 4 Discussion

### 4.1 Principal findings and policy interpretation

The adult WHO-reported coverage indicator remained below the corresponding SAC indicator in every year from 2014 to 2024, under every prespecified data-quality exclusion, and among a fixed set of 48 countries with valid age-specific denominators throughout. In 2024, the adult indicator was 14.6% and the SAC indicator was 61.7%. The more informative result was their symmetric decomposition. Adult reporting support and conditional reported coverage were both lower than the corresponding SAC values: 47.7% versus 85.2%, and 30.6% versus 72.3%, respectively. A single age-group ratio therefore combined the proportion of the denominator linked to valid treatment information with the level of treatment reported within supported records.

This persistence has different meanings across policy eras. The 2014–2021 series documents the reporting architecture inherited from a SAC-centred control strategy; earlier recommendations included selected at-risk adults, but did not create the same adult-treatment expectation as the 2022 recommendation for all age groups from 2 years of age in qualifying communities. The 2022–2024 observations are only early calendar years at or after publication of the broader WHO recommendations (2, 11). National adoption and implementation dates were unavailable, and the transition overlapped COVID-era disruption (31). The study therefore provides a baseline for the reporting architecture and an early diagnostic assessment for monitoring, not an estimate of guideline impact.

The adult denominator partition is important for this interpretation. In 2024, 34.3% was associated with SAC-only records and 18.0% with records without an age label. The former may describe a programme whose scope was restricted to SAC; the latter leaves age-specific information unresolved. The observed adult reporting-denominator share of 47.7% therefore measures linked treatment information, not the percentage of an expected adult programme that was successfully reported. Establishing a reporting deficit would additionally require national eligibility rules, campaign scope, and expected submission fields.

Most importantly, the reported-indicator difference is not an identified treatment-receipt difference. The 2024 logical missing-report range extended from −61.8 to +5.3 percentage points and included zero. The direction of the difference in actual treatment receipt between adults and SAC could therefore reverse under an extreme but arithmetically admissible allocation of unreported treatment. The firm conclusion is that fewer adult treatments were visible relative to the full valid adult requirement denominator—not that the public compilation proves that a lower proportion of adults actually swallowed praziquantel.

### 4.2 Programme-monitoring implications

The findings join three research streams. Parasitological and modelling work explains why a SAC-centred strategy may be insufficient where adults contribute materially to infection, morbidity, or transmission (4–6). Implementation studies show that adult treatment depends on community delivery, knowledge, opportunity costs, perceived need, and concerns about adverse effects (7–10). Routine-information research shows that technical definitions, completeness, organizational routines, and use in decision-making jointly determine the value of a reporting system (12–17). Our analysis links these streams in the context of monitoring: the source must first reveal what information is available before a global ratio can be used to assess treatment delivery.

The decomposition supplies a diagnostic sequence rather than a causal mechanism. A low reporting-denominator share calls first for an audit of programme eligibility, age-specific data capture, submission status, and preservation of age fields during aggregation. Low conditional reported coverage calls for investigation of medicine availability, community distribution, mobilization, accessibility, and acceptance. If both are low, data processes and implementation require attention together. Table 5 assigns these questions to the WHO and data stewards, national neglected tropical disease (NTD) programmes, and local implementation teams without assuming which mechanism occurred in any country.

**Table 5:** Action matrix for age-specific reporting diagnostics.

Diagnostic state	WHO and global data stewards	National NTD programme	District/local implementation and validation
Low reporting-denominator share	Publish reporting completeness beside coverage; use mutually exclusive age fields; version data dictionaries; preserve the distinction between zero and missing values, and retain submission-reason codes	Reconcile age-specific eligibility, denominators, treatment counts, and submission status before national aggregation	Verify whether missing adult information reflects programme scope, forms, transmission, or aggregation loss
Low conditional reported coverage	Flag numerator–denominator inconsistencies and values > 100%; retain annotated revisions	Audit medicine availability, campaign design, community channels, and aggregation of treatment registers	Investigate non-reach, non-participation, accessibility, timing, and acceptance; use post-MDA surveys where needed
Both components low	Require a joint data-quality and implementation review rather than interpreting the final ratio alone	Coordinate data, supply, and delivery teams; document corrective actions	Pair register verification with coverage evaluation and qualitative implementation inquiry
Minimum future age standard	Separate denominators and treatment numerators for preschool-age children, SAC, and adults; align reporting periods and geographic units	Record the eligibility rule, campaign status, reason for zero or missing values, and revision history	Retain sufficiently granular data to detect local heterogeneity while protecting confidentiality

WHO, World Health Organization; NTD, neglected tropical disease; MDA, mass drug administration; SAC, school-age children.

Independent evidence is necessary after that first diagnostic step. Across NTD campaigns, programme-reported and survey estimates have disagreed often enough to change threshold-based decisions (21). Schistosomiasis coverage surveys in Ethiopia and Zanzibar separated being offered treatment from swallowing it and identified reasons for non-reach or non-participation (10, 22). More granular analysis in Mozambique showed that district summaries can conceal subdistrict variation (23); programme assessments in Ghana and Sierra Leone showed both the value and the limits of combining intervention histories, prevalence surveys, and coverage monitoring (24, 25). The present country-year analysis cannot replace those designs. It indicates when global reporting cannot support the local inference being requested.

### 4.3 Bounded health-data-equity interpretation

The relevance to equity lies in the ability to assess eligible age groups using comparably explicit information. In this study, age-group visibility was operationalized through the denominator share linked to valid treatment reports and the adult information-state partition. The principle of data equity explains why comparable representation matters for monitoring and corrective decisions (15); it does not label every difference in those measures as inequitable. A complete report from a legitimately SAC-only programme can produce low adult visibility without an adult reporting failure, whereas loss of adult information from an adult-inclusive programme is a different problem. The public compilation cannot distinguish these situations without eligibility and programme-scope metadata. Nor does comparable data representation require equal coverage percentages across groups with different treatment needs. The study measured neither individual denial of care nor inequality by sex, socioeconomic position, geography, disability, or their intersections; the 47.1-percentage-point reported-indicator difference is not an individual health-equity estimate.

### 4.4 Preschool-age visibility under an all-age policy

Preschool-age children illustrate a second form of limited visibility: a policy-relevant group may lack a separately identifiable category, rather than merely have fewer valid reports. The WHO’s 2022 recommendation includes children from 2 years of age, including those aged 2–4 years outside the source’s SAC definition (2, 11, 19). This workbook did not separately identify their treatment, so neither preschool-age reported coverage nor reporting-denominator share could be estimated. Their absence as a distinct analytical category is not evidence that no preschool-age child was treated. It shows why SAC/adult monitoring cannot establish implementation of an all-age recommendation. Future reporting should distinguish preschool-age requirement and treatment counts, with explicit age limits and eligibility rules, alongside the corresponding counts for SAC and adults. Adding a preschool-age field would also require revising the residual-based adult derivation rather than carrying the present subtraction forward unchanged.

### 4.5 Source validity, reproducibility, and transferability

The adult derivation was supported for this frozen source by the WHO’s SAC/adult denominator model, explicit workbook labels, historical reporting documentation, and concordance with the WHO’s public 2024 aggregation (18, 19, 29, 30, 32). Concordance verified our implementation of the source logic; it did not independently validate submitted country values. Four records labelled Adults and all arithmetic anomalies were retained for record-level audit. A future source version containing an All label or a separate preschool-age field would cause the present workflow to stop and would require a revised data model. The 2022 guideline includes children from 2 years of age, so the policy population remains broader than the observed SAC/adult reporting construct.

The reproducible contribution is the combination of practices rather than the elementary factorization itself: frozen source bytes, a source-specific semantic contract, explicit missing-versus-zero states, symmetric decomposition, state partitioning, prespecified anomaly handling, fixed-support and influence analyses, external aggregation anchors, and clean-room verification. This workflow identifies what the reporting system makes visible, what observable reporting states shape that visibility, and which conclusions remain unsupported. Transfer to another NTD or database would require fresh validation of age categories and count relationships. The computation is portable; the source semantics are not.

### 4.6 Strengths and limitations

The strengths of this study included complete preservation of the public country-year source, explicit field derivations, prespecified anomaly rules, symmetric age-group accounting, assessment of sensitivity to country composition, and machine-tested reproduction. The study also avoided sampling intervals that would mischaracterize systematic reporting uncertainty as sampling error.

Several limitations remain. First, reported treatment did not constitute independently validated uptake, and the reports contained no information about adherence, biological effect, infection intensity, or morbidity. Second, missing treatment information could represent zero treatment, unreported treatment, a SAC-only plan, or another state that country-year data could not adjudicate. Logical envelopes demonstrated this non-identification but were deliberately extreme, not statistical uncertainty intervals. Third, adult counts inherited any error or time-reference mismatch in total and SAC components, and denominators may have been revised. Fourth, population-weighted aggregates described neither the average endemic country nor within-country heterogeneity. Fifth, only three calendar years in the analysis fell at or after guideline publication, and national implementation timing was unavailable. Sixth, preschool-age children were not separately identifiable in the source, preventing evaluation of the full age range covered by the 2022 recommendation. Finally, semantic and computational verification could not substitute for country-level validation of submitted values.

## 5 Conclusion

The WHO country reports showed a lower reported-coverage indicator for adults than for SAC in each year during 2014–2024, spanning an inherited SAC-centred policy period and early years after broader guideline publication. In 2024, both the adult denominator share linked to valid treatment information and conditional reported coverage were lower than the corresponding SAC components. The information-state partition shows why low adult visibility may combine programme scope and incomplete reporting, and the logical missing-report ranges do not establish the actual ordering of treatment coverage between adults and SAC. Preschool-age children were not separately observable despite their inclusion in the broader policy. Age-inclusive monitoring requires explicit age-specific counts, documented eligibility and reporting states, and auditable revisions, supported where needed by independent coverage surveys and local implementation inquiry.

## Data Availability

Publicly available data were analyzed in this study. The study uses aggregate country-year data from the WHO PCT Databank—Schistosomiasis, available at https://www.who.int/teams/control-of-neglected-tropical-diseases/data-platforms/pct-databank/schistosomiasis. Because the live WHO workbook may be revised, the downloaded snapshot was archived. The merged editor- and reviewer-only workbook contains the source cell values, a recoverable copy of the original WHO workbook, analysis code, pinned environment specifications, numerical results, automated tests, technical verification information, and reproduction instructions. File checksums are provided in the workbook documentation. The separate reproduction files are also available from the corresponding author on reasonable request. No new data were collected.
